# Establishment of a low-temperature immersion method for extracting high-activity and high-purity mitochondria from *Syntrichia caninervis* Mitt.

**DOI:** 10.1186/s13007-025-01419-z

**Published:** 2025-07-26

**Authors:** Wenting Huo, Xiaohua Lin, Mengyu Gao, Xiang Shi, Hongbin Li, Lu Zhuo

**Affiliations:** 1 Key Laboratory of Xinjiang Phytomedicine Resource and Utilization of Ministry of Education, Key Laboratory of Oasis Town and Mountain-basin System Ecology of Xinjiang Production and Construction Corps, College of Life Sciences, Shihezi University, Shihezi, 832003 China; 2 College of Agriculture, Shihezi University, Shihezi, 832000 China

**Keywords:** *Syntrichia caninervis* Mitt., Mitochondria isolation, Low-temperature immersion method, Activity, Storage time

## Abstract

**Background:**

Mitochondria are central to plant growth, development, and stress resilience. Despite their importance, mitochondrial research in desiccation-tolerant mosses remains underexplored. To unravel the stress resistance mechanisms of the extremotolerant desert moss, establishing a method to isolate highly active and pure mitochondria is critical. This study pioneered the use of low-temperature immersion combined with differential centrifugation and discontinuous percoll density gradient centrifugation to isolate mitochondria from *Syntrichia caninervis*, a model desiccation-tolerant moss. The purity, structural integrity, and functional activity of the isolated mitochondria were systematically evaluated using western blot analysis, Janus Green B staining, JC-1 membrane potential assays, and electron transport chain (ETC) complex activity measurements.

**Results:**

From 50 g of *S. caninervis* tissue, approximately 56.7 mg of mitochondria were isolated with high purity, effectively removing non-mitochondrial contaminants (e.g., chloroplasts and cytoplasmic debris). Functional assays and membrane potential analysis confirmed no significant damage to mitochondrial activity or structural integrity during the purification process. Notably, room temperature storage (25 °C) induced rapid functional decay, whereas cryogenic storage at − 20 °C maintained ≥ 70% mitochondrial viability over 10 days, sufficient for downstream applications including proteomic profiling and bioenergetic studies.

**Conclusion:**

The optimized mitochondrial isolation protocol presented here is both time efficient and highly reproducible, yielding mitochondria of exceptional purity suitable for mechanistic studies in desiccation-tolerant mosses. The isolated mitochondria exhibit robust functional activity and structural integrity, providing a reliable platform for investigating stress resistance mechanisms in *S. caninervis* and other extremophytic species. By establishing a standardized workflow for mitochondrial isolation in desiccation-tolerant plants, this method addresses a critical technical gap and paves the way for advanced investigations into mitochondrial biology under extreme environmental conditions.

## Background

*Syntrichia caninervis* Mitt. (Pottiaceae: Syntrichia), a desiccation-tolerant moss widely distributed in the Gurbantunggut Desert of Xinjiang, China, serves as a dominant species in biological soil crusts [1, 2]. This extremophytic moss exhibits exceptional stress tolerance, surviving over 98% cellular dehydration (anhydrobiosis), cryogenic freezing (− 196 °C), and gamma radiation exposure exceeding 5000 Gy [3]. These traits make it a promising candidate for pioneering plant colonization in extraterrestrial environments such as Mars. Recent research on *S. caninervis* has focused on its growth distribution [4, 5], physiological-biochemical characteristics [6–8], dehydration-rehydration stress mechanisms, and stress-responsive gene identification [9–11]. Studies highlight its unparalleled stress resistance compared to other terrestrial plants, positioning it as an ideal model for investigating plant adaptation to extreme desert habitats and evolutionary transitions [3, 12].

Mitochondria, the primary cellular hubs for metabolism and energy production, generate over 90% of adenosine triphosphate (ATP) required for organismal growth, development, and reproduction [12, 13]. Beyond energy provision, these organelles play multifaceted roles in stress signaling, redox homeostasis, and programmed cell death [14, 15]. Emerging evidence indicates that plant mitochondria act as integrators of environmental stress cues, transducing abiotic signals into cellular responses through dynamic changes in membrane potential (ΔΨm). Altered mitochondrial ultrastructure and matrix electron density often precede stress-induced changes in other organelles [16–18]. Our preliminary studies revealed that *S. caninervis*, a desiccation-tolerant moss, maintains intact mitochondrial cristae structure under extreme heat stress. Comparative transcriptomic analysis identified 8.25% of differentially expressed genes (DEGs) associated with mitochondrial energy metabolism (unpublished data). While mitochondrial isolation protocols for higher plants (e.g., Arabidopsis, citrus) exist, they are suboptimal for bryophytes like *S. caninervis* due to fundamental differences in cellular architecture and stress physiology [18]. Bryophytes possess robust cell walls with unique polysaccharide compositions and highly resilient membrane systems under desiccation, which render traditional mechanical homogenization or enzymatic digestion methods ineffective [18, 19]. Unlike vascular plants, desiccation-tolerant bryophytes require gentle disruption methods to preserve mitochondrial integrity during hydration-rehydration cycles. Therefore, these findings suggest a central role for mitochondria in the extreme stress resilience of *S. caninervis*, nevertheless, critical mechanistic questions remain: (a) What regulatory pathways enable mitochondria to maintain ATP production during dehydration, heating, and irradiation?; (b) How do mitochondrial ultrastructural and functional adaptations contribute to stress tolerance? Answering these requires robust experimental systems. However, the specific molecular mechanisms underlying its stress resistance remain poorly understood, and these questions require further investigation through comprehensive and in-depth studies. Critically, successful isolation of highly active and high-purity mitochondria from *S. caninervis* represents an essential prerequisite for advancing these mechanistic investigations.

This study presents an advanced mitochondrial isolation method tailored for desiccation-tolerant mosses, enabling the extraction of high-purity, functional mitochondria from *S. caninervis*. The protocol employs low-temperature immersion (4 °C, 8–12 h) treatment to minimize chemical/enzymatic damage to cellular structures while reducing operational time compared to traditional isolation methods. The extraction buffer contains protective additives, including phenylmethylsulfonyl fluoride (PMSF, a protease inhibitor), polyvinylpyrrolidone (PVP, a phenolic compound binder), and sodium ascorbate (an antioxidant). Mitochondrial purity, structural integrity, and functional activity were systematically validated through sequential differential centrifugation and discontinuous percoll density gradient centrifugation. Of course, to confirm the purity of the isolated plant mitochondria, we evaluated the extract concentration at the protein level using western blot analysis. Mitochondrial ultrastructural integrity was dual-validated through confocal laser scanning microscopy with MitoTracker-specific fluorescent staining and ultrathin-section transmission electron microscopy (TEM) analysis. Additionally, mitochondrial membrane potential and the functional activity of inner membrane electron transport chain (ETC) complexes were assayed to ensure the suitability of the mitochondria for subsequent studies. Specifically, we assessed the temporal changes in activity and integrity of mitochondria isolated from *S.caninervis* under standard laboratory temperature conditions. This study establishes a critical material foundation for subcellular-level investigations into the stress resistance physiology and molecular biology of mitochondria in moss plant cells.

## Results

### Isolation of mitochondria

**Fig. 1:**
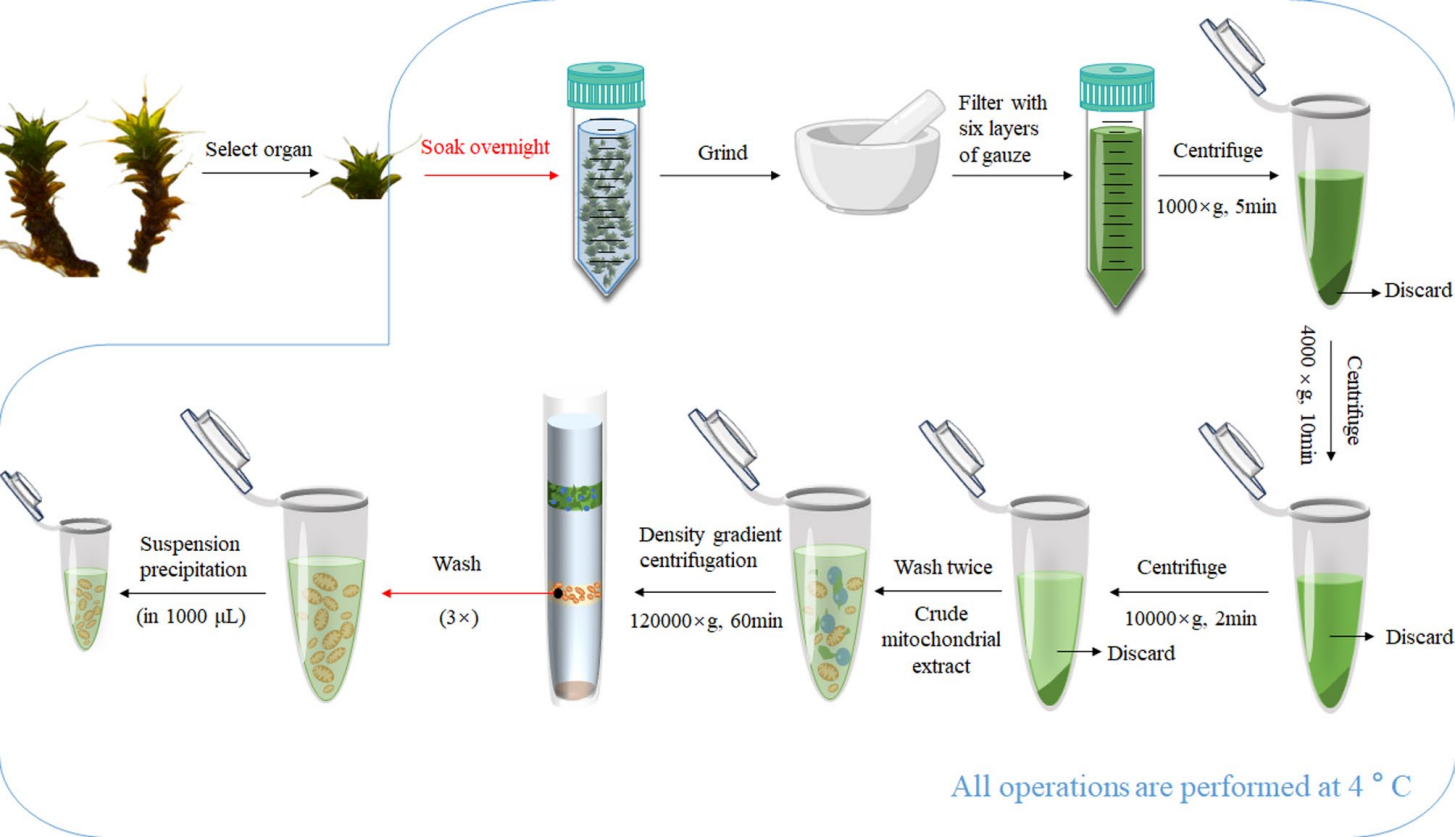
Procedure for mitochondria isolation from *Syntrichia caninervis* Mitt

The low-temperature immersion treatment allows the extraction buffer to fully diffuse into intercellular spaces, enabling gradual mitochondrial release via osmotic gradients while minimizing enzymatic damage caused by homogenization, thereby effectively preserving mitochondrial morphology. In both the extraction and suspension buffers, sucrose (traditionally used to maintain osmotic balance) was replaced with sorbitol, whose viscosity and density provide stable physical support to mitochondria, reducing collisions and aggregation during suspension. The extraction and suspension buffers were supplemented with the following components: HEPES (4-(2-hydroxyethyl)-1-piperazineethanesulfonic acid): A zwitterionic buffering agent that stabilizes the pH of the suspension, ensures proper enzymatic function in mitochondria, and improves the compatibility of the mitochondrial suspension buffer. Crucially, HEPES does not chelate Mg²⁺/Ca²⁺ ions, thereby preserving the activity of mitochondrial respiratory chain enzymes. Polyvinylpyrrolidone (PVP): Adsorbs phenolic compounds released from plant tissues, preventing their interference with mitochondrial integrity. Sodium ascorbate: reduces mitochondrial aggregation and protects mitochondrial membrane lipids from oxidative damage by acting as an antioxidant. Finally, discontinuous percoll density gradient centrifugation was employed to remove chloroplasts and nuclei, yielding purified mitochondria (Fig. 1). Building on prior mitochondrial isolation protocols, this study optimized the extraction buffer and centrifugation protocol, thereby significantly enhancing the mitochondrial extraction efficiency for *S. caninervis*.

### Mitochondrial production and purity

**Fig. 2:**
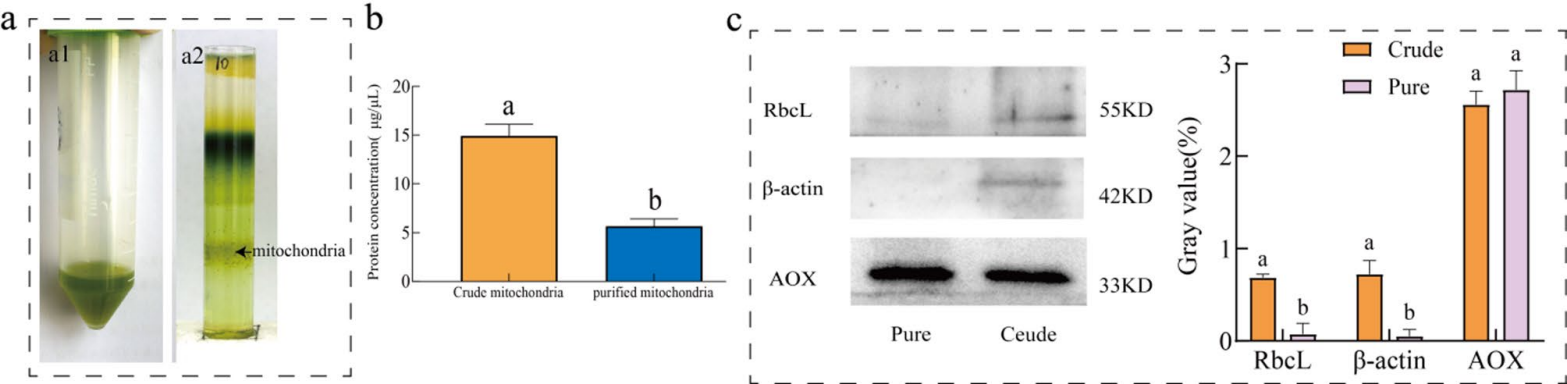
Validation of mitochondrial yield and purity in *Syntrichia caninervis* Mitt. (**a**). Appearance of the centrifugal tubes. **a1**: Crude mitochondrial extract resuspension. **a2**: Layered separation after Percoll density gradient centrifugation. (**b**). Mitochondrial yield. Comparison of protein content between crude and purified mitochondrial extracts. Error bars represent the standard deviation of the mean (*n* = 3). One-way ANOVA was used to determine significant differences: identical lowercase letters indicate no significant difference, while different letters denote significant differences (significance level α = 0.05).(**c**). Mitochondrial purity validation. Purity was confirmed via Western blot using AOX (mitochondrial marker), Rubisco (chloroplast marker, RbcL), and β-actin (cytoplasmic marker). Grayscale value analysis (*n* = 3) demonstrated enriched mitochondrial AOX and negligible contamination from chloroplasts/cytoplasm. Error bars show the standard deviation of the mean. Different lowercase letters indicate significant differences (α = 0.05)

Bicinchoninic Acid Assay (BCA) analysis revealed that the total protein content of the isolated mitochondria was 5.67 ± 0.61 µg/µL, accounting for 35.87% of the crude mitochondrial extract (14.93 ± 0.96 µg/µL) (Fig. 2a and b). To assess mitochondrial purity at the protein level, western blot was performed using AOX (alternative oxidase) as the mitochondrial marker, RbcL (ribulose-1,5-bisphosphate carboxylase/oxygenase) as the chloroplast marker, and β-actin as the cytoplasmic marker. Western blot analysis showed that the band intensity of AOX in purified mitochondria was slightly higher than that in the crude extract (Fig. 2c). Conversely, the band intensities for RbcL (chloroplast-specific) and β-actin (cytoplasmic) in purified mitochondria were negligible and significantly lower than those in the crude extract (*P* < 0.01). These results confirm that percoll density gradient centrifugation is highly effective for isolating high-purity mitochondria from the desiccation-tolerant moss *S.caninervis*. Furthermore, approximately 56.7 mg of mitochondria were obtained from 50 g of moss tissue, providing sufficient experimental material for subsequent mechanistic studies on mitochondrial stress tolerance.

### Assessment of mitochondrial integrity and membrane potential

**Fig. 3:**
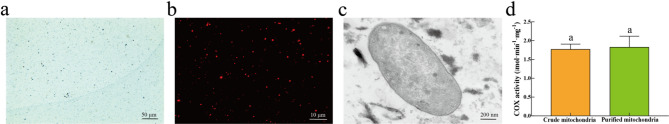
Assessment of mitochondrial activity and morphology. (**a**). Mitochondria stained with the mitochondria-specific dye JG-B, observed via bright-field microscopy. (**b**). Mitochondria stained with the fluorescent dye MitoTracker Red, visualized using fluorescence microscopy. (**c**). Mitochondrial ultrastructure analyzed by transmission electron microscopy (TEM). (**d**). Cytochrome c oxidase (COX) activity measured as the decrease in absorbance at 550 nm due to oxidation of reduced cytochrome c. Activity is expressed as µmol cytochrome c oxidase/min/mg mitochondrial protein. Error bars represent the standard deviation of the mean (*n* = 3). One-way ANOVA was used to determine significant differences: identical lowercase letters indicate no significant difference, while different letters denote significant differences (α = 0.05). Student’s t-test was applied for pairwise comparisons

Mitochondria typically exhibit four morphological states: punctate, vermicular, giant, and diffuse. Among these, punctate mitochondria (small and round or short rod-shaped) are indicative of high functional activity, while vermicular, giant, and diffuse morphologies are often associated with stress or dysfunction. In this study, isolated mitochondria were stained with the mitochondrial-specific dyes JG-B (Fig. 3a) and MitoTracker (Fig. 3b), revealing mitochondria as variably sized granules or short rods. Transmission electron microscopy (TEM) further confirmed the structural integrity of isolated mitochondria (Fig. 3c). Additionally, cytochrome c oxidase (COX)—a large transmembrane protein complex embedded in the mitochondrial inner membrane—ensures efficient oxygen utilization during aerobic respiration by catalyzing the reduction of molecular oxygen. Hypoxia or low-oxygen conditions impair COX function, and prolonged hypoxia can lead to cellular dysfunction or death. COX activity assays demonstrated minimal variation between crude (1.77 ± 0.11 µmol/min/mg) and purified (1.83 ± 0.24 µmol/min/mg) mitochondrial extracts (Fig. 3d), confirming that the isolated mitochondria retained intact respiratory function without sustaining damage. These results collectively indicate that the optimized low-temperature immersion method successfully yielded highly active, high-purity mitochondria from *S. caninervis* cells, with preserved structural and functional integrity.

**Fig. 4:**
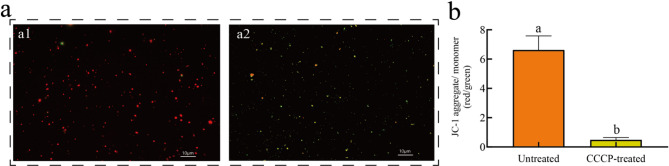
Mitochondrial membrane potential in *Syntrichia caninervis* Mitt. (**a**). Fluorescence imaging of mitochondrial membrane potential in isolated mitochondria. **a1**: JC-1 staining of untreated mitochondria showing red fluorescence (JC-1 aggregates), indicating active mitochondria with high ΔΨm. **a2**: JC-1 staining of CCCP-treated mitochondria showing green fluorescence (JC-1 monomers), indicating loss of membrane potential (ΔΨm) and mitochondrial inactivity. (**b**). Quantitative analysis of mitochondrial membrane potential. The JC-1 aggregate/monomer ratio was significantly higher in untreated mitochondria (6.62 ± 0.79) compared to CCCP-treated mitochondria (0.48 ± 0.13, *P* < 0.01). Data represent mean ± SD (*n* = 3)

Mitochondrial membrane potential (ΔΨm) was assessed using the fluorescent probe JC-1 (5,5′,6,6′-tetrachloro-1,1′,3,3′-tetraethylbenzimidazolylcarbocyanine iodide). JC-1 selectively accumulates in functional mitochondria with high ΔΨm, forming red fluorescent aggregates, whereas in depolarized (low ΔΨm) or apoptotic mitochondria, it remains as green fluorescent monomers. Following JC-1 staining, fluorescence microscopy revealed that isolated mitochondria were predominantly labeled with punctate red fluorescence (Fig. 4a1). In contrast, treatment with the mitochondrial uncoupler CCCP abolished mitochondrial activity, resulting in green punctate fluorescence (Fig. 4a2). ImageJ software was used to quantify the JC-1 aggregate/monomer ratio (Fig. 4b). Untreated mitochondria exhibited a significantly higher ratio (6.62 ± 0.79) compared to CCCP-treated mitochondria (0.48 ± 0.13, *P* < 0.01). These results further confirm the reliability of the optimized protocol for isolating high-activity mitochondria from *S. caninervis*.

### Activity of mitochondrial inner membrane complexes

**Fig. 5:**
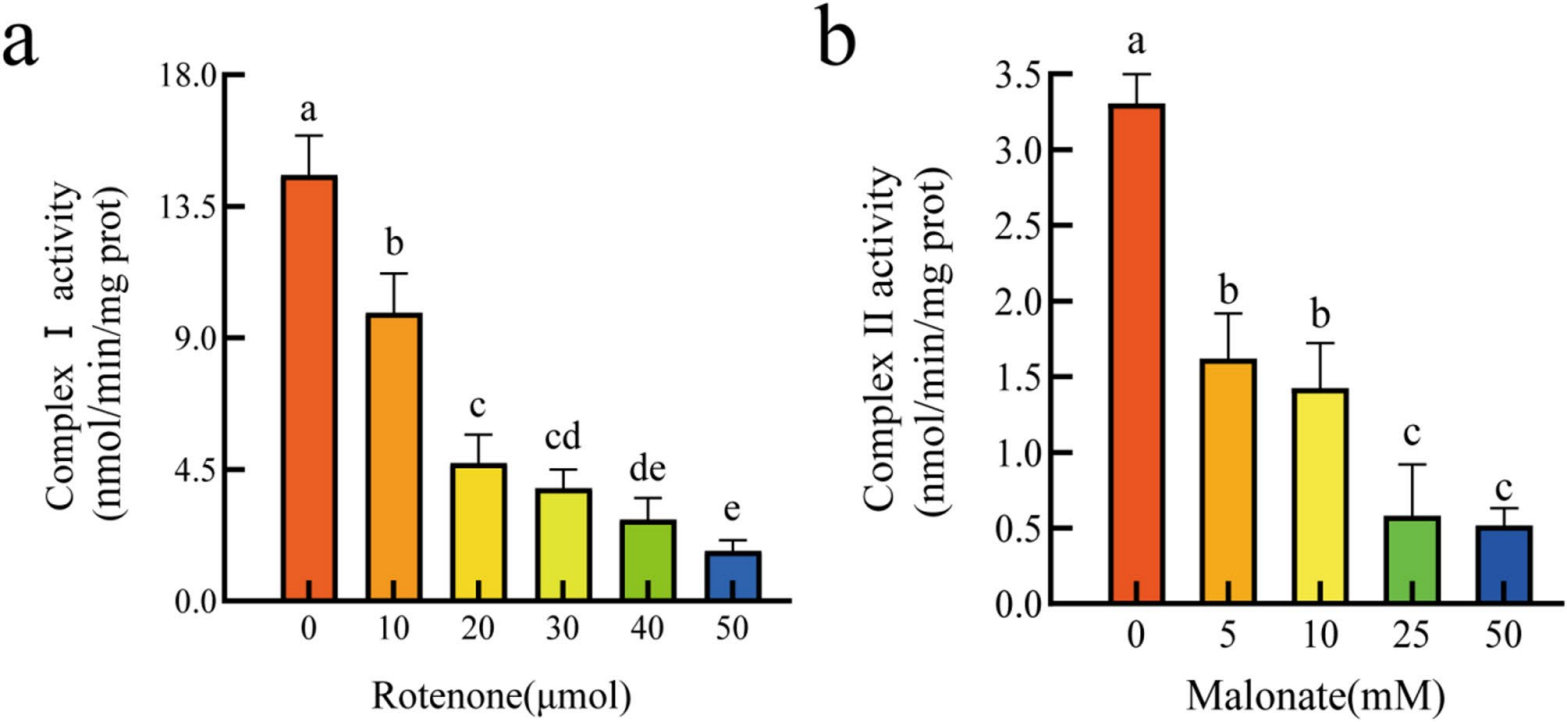
Activity assays of mitochondrial inner membrane complexes. (**a**). Complex I activity was measured following treatment with the specific inhibitor rotenone (0–50 µM). (**b**). Complex II activity was assessed using the specific inhibitor malonate (0–50 mM). Error bars represent the standard deviation of the mean (*n* = 3). One-way ANOVA was used to determine significant differences: identical lowercase letters indicate no significant difference, while different letters denote significant differences (α = 0.05)

The activity of electron transport chain (ETC) complexes is a critical indicator of energy metabolism in organisms, and their functional state reliably reflects the structural integrity of mitochondria. To validate the structural and functional integrity of isolated mitochondria, this study employed graded concentrations of inhibitors targeting specific ETC complexes. The results demonstrated that both rotenone (0–50 µM), a specific inhibitor of mitochondrial complex I, and malonate (0–50 mM), a competitive inhibitor of succinate dehydrogenase (complex II), significantly suppressed the activity of the inner mitochondrial membrane electron transport chain complexes. Compared to the control (0 µM rotenone), 30 µM rotenone reduced Complex I activity to 26.47 ± 0.02% (*P* < 0.001), while 50 µM rotenone further decreased activity to 11.76 ± 0.02% (*P* < 0.001) (Fig. 5a). For malonate, inhibition of Complex II was more pronounced: 5 mM malonate retained 49.09 ± 0.07% activity (*P* < 0.001), whereas 25 mM and 50 mM treatments reduced activity to 17.67 ± 0.08% and 15.71 ± 0.03% (*P* < 0.001) (Fig. 5b), respectively. Notably, at equivalent concentration gradients (e.g., 50 µM vs. 50 mM), rotenone exhibited significantly higher inhibitory efficiency against Complex I (88.24% activity loss) compared to malonate against Complex II (84.29% activity loss), suggesting greater sensitivity of Complex I to its inhibitor. This phenomenon indicates that the isolated mitochondria retain an intact inner membrane system capable of sensitive and predictable responses to exogenous inhibitors, preserving both structural stability and enzymatic regulatory capacity of oxidative phosphorylation machinery. Collectively, these data confirm that the isolated mitochondrial preparations exhibit high functional activity, fulfilling the requirements for downstream mechanistic studies.

### Mitochondrial activity over time under different temperatures

**Fig. 6:**
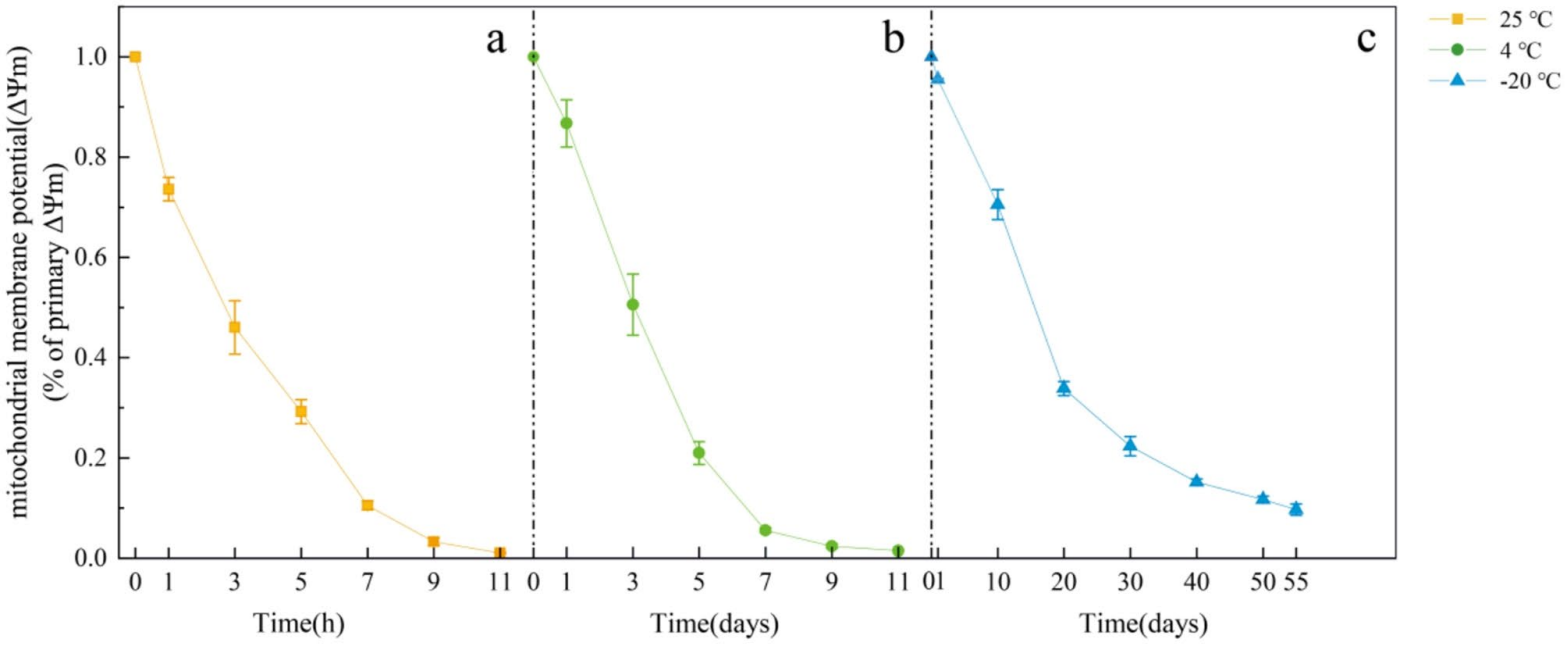
Changes in mitochondrial activity of *Syntrichia caninervis* Mitt. under different temperatures (25 ℃, 4 ℃, and − 20 ℃). (**a**). Changes of mitochondrial activity at 25℃. (**b**). Changes of mitochondrial activity at 4 ℃. (**c**). Changes of mitochondrial activity at -20 ℃. Error bars represent the standard deviation of the mean (*n* = 3)

This study examined the time-dependent changes in mitochondrial activity under commonly used laboratory storage temperatures (25℃, 4℃, and − 20℃). The results revealed that the isolated mitochondria retained 80% of their initial activity after 0.89 h at 25℃ (Fig. 6a), 1.52 days at 4℃ (Fig. 6b), and 8.65 days at -20℃ (Fig. 6c). Activity declined to 50% of the control levels at 2.93 h (25℃), 3.06 days (4℃), and 16.86 days (-20℃), and further decreased to 30% at 4.98 h (25℃), 4.45 days (4℃), and 23.8 days (-20℃). Complete loss of activity occurred after 5 h at 25℃, 6 days at 4℃, and 60 days at -20℃. These findings indicate that room temperature (25℃) storage should be avoided due to rapid activity degradation, while short-term preservation at 4℃ (up to 6 days) is feasible. Notably, -20℃ storage significantly extended mitochondrial viability (up to 60 days), demonstrating superior performance for long-term experimental applications compared to other temperatures.

## Discussion

*S. caninervis*, a dominant species in biological soil crusts of the Gurbantunggut Desert, exhibits exceptional stress resistance and has emerged as a model organism for studying desiccation tolerance mechanisms [2, 3]. Mitochondria, as the energy metabolism hub of the cell, are intrinsically linked to stress tolerance mechanisms. They play a central role in reactive oxygen species (ROS) scavenging, energy supply, and signal transduction, which are critical for cellular adaptation to environmental stresses [15, 16]. Establishing an efficient protocol for isolating highly pure mitochondria from *S. caninervis* is critical for mechanistic investigations into its stress resistance and provides a theoretical foundation for understanding desert plant adaptation strategies. Building upon established methods for higher plants [19–25], this study optimized extraction buffer composition, pH, and centrifugation parameters (speed, duration) to develop a novel protocol combining cryogenic maceration with differential centrifugation and discontinuous percoll density gradient centrifugation. This study establishes a standardized protocol for *S. caninervis* while simultaneously providing an adaptable technical framework for subcellular research in other desiccation-tolerant moss. The parameter optimization strategy presented here may facilitate comparative investigations of mitochondrial adaptation mechanisms across extremophiles, ultimately advancing our understanding of universal principles governing organelle-level stress responses.

To obtain high-quality mitochondria, the isolation protocol was tailored to the unique cellular structural features of the desiccation-tolerant moss *S. caninervis*, such as its robust cell wall toughness, mechanical strength, and intact membrane system integrity [26–28]. Prior to experimental procedures, moss tissues were subjected to cold maceration (4℃, 8–12 h) in an optimized extraction buffer. The low temperature reduced the fluidity of the phospholipid bilayer in cell membranes and inhibited enzymatic activity [29, 30], while maceration allowed the tissues to fully equilibrate with the isotonic extraction buffer, enabling the gradual release of mitochondria and minimizing membrane damage caused by mechanical disruption. This approach ensured enhanced structural stability of the isolated mitochondria [31]. Undoubtedly, during high-speed centrifugation, mitochondria are highly susceptible to structural damage, which significantly compromises experimental outcomes [32]. To address this, we systematically compared three traditional density gradient centrifugation methods (sucrose, percoll, and nycodenz) [21–23, 33, 34], Our results demonstrate that percoll density gradient centrifugation is the optimal method for mitochondrial enrichment in mosses. Through systematic optimization, we established a three-step discontinuous gradient with concentrations of 23%, 35%, and 42% percoll, which yielded mitochondria with higher purity and activity compared to commercial extraction kits. Notably, while previous studies using percoll gradient purification yielded 1.2 mg of mitochondria from 50 g of *Arabidopsis* leaves [35], our protocol achieved ~ 56.7 mg of mitochondria from just 50 g of *S. caninervis* tissue. It can be concluded that the pretreatment of low-temperature immersion, combined with differential centrifugation and discontinuous percoll density gradient centrifugation, significantly increased the mitochondrial content of *S. caninervis*, and this method greatly reduced the manual operation time.

Furthermore, this study established a standardized multi-dimensional validation system to comprehensively assess mitochondrial purity, integrity, and activity. MitoTracker, as a mitochondria-targeting fluorescent probe, utilizes its cationic properties to actively accumulate within mitochondria through membrane potential [36]. When combined with the three-dimensional reconstruction capability of confocal laser scanning microscopy (CLSM), it enables clear visualization of mitochondrial morphology. Ultrathin-section transmission electron microscopy (TEM) of *S. caninervis* mitochondria confirmed intact cristae architecture and double-membrane structures at nanoscale resolution, significantly outperforming traditional methods in ultrastructural analysis. This approach provides for future investigations into mitochondrial stress resistance mechanisms at the subcellular level. Furthermore, CCCP treatment induced a 13-fold reduction in mitochondrial membrane potential (ΔΨm) compared to untreated controls. Concurrently, all tested inner membrane electron transport chain (ETC) complexes exhibited decreased activity, indicates that the functional activity of mitochondrial electron transport chain (ETC) complexes was inhibited, confirming that mitochondrial activity remained preserved during the extraction process. These findings collectively demonstrate that the method effectively ensures mitochondrial purity, integrity, and viability, thereby enabling its application in subsequent physiological and biochemical analyses.

This study systematically evaluated mitochondrial activity kinetics under common laboratory storage conditions, providing practical guidance for selecting appropriate preservation temperatures and durations based on experimental needs. Notably, isolated mitochondria exhibited rapid functional decay at room temperature (25 °C), necessitating immediate use or cryogenic storage. Conversely, mitochondria stored at − 20 °C maintained ≥ 70% functional viability for 10 days, sufficient for most downstream applications, including proteomic profiling, bioenergetic assays, and stress-response studies. These findings underscore the protocol’s adaptability to diverse research requirements while emphasizing the critical importance of controlled temperature storage for preserving mitochondrial integrity and activity. Additionally, this work establishes a foundational framework for studying plant mitochondrial responses to abiotic stress, with implications for agricultural biotechnology and arid-region conservation.

## Conclusion

This study first established a comprehensive and efficient mitochondrial isolation protocol for *S. caninervis* by combining low-temperature soaking pretreatment with differential centrifugation and discontinuous percoll density gradient centrifugation. Furthermore, we systematically evaluated the yield, integrity/activity of isolated mitochondria, and their activity kinetics under varying temperatures using western blot, Janus Green B staining, and JC-1 membrane potential assays—marking the first application of this integrated analytical framework in bryophyte mitochondrial studies. These findings provide valuable references for high-quality mitochondrial isolation in moss plants and establish an exemplary model for assessing mitochondrial purity, integrity, and activity in non-model organisms.

## Materials and methods

### Plant materials

In December 2024, small crust fragments (2 × 2 cm) of *S. caninervis* with consistent growth were collected from the Gurbantunggut Desert (44°62′N, 88°26′E) in Xinjiang, China. Samples were collected every 5 m, totaling 10 samples, which were placed in kraft paper bags along with their soil matrix and transported to the laboratory. The samples were stored under low-light conditions, low humidity (~ 20%), and a controlled temperature of 20 ± 1℃. Prior to experiments, intact *S. caninervis* plants were rehydrated by immersion in distilled water in a tray and incubated in a growth chamber (20 ± 1℃) under a 16 h/8 h light-dark cycle for 72 h, followed by 48 h in a dark chamber (20 ± 1℃). Mature individuals of similar size were selected from the crust fragments (50 g), and their rhizoids were trimmed with scissors. The samples were rinsed with distilled water, blotted dry with filter paper, and placed in 50mL centrifuge tubes for further use.

### Isolation of crude mitochondria (Differential centrifugation)

50 g of *S caninervis* material were homogenized with 30mL of extraction buffer (25mM HEPES, 2mM caproic acid, 0.4 M sorbitol, 2mM DTT, 5mM EDTA, 15mM Na-AsA, 1mM PMSF, 1% PVP, 0.5% BSA, pH 7.8) in a centrifuge tube at 4℃ for 8–12 h. The homogenate was filtered through six layers of gauze, and the filtrate was collected. The homogenate was centrifuged at 4℃ (Hitachi CR22N/40A/3 high-speed centrifuge, Japan) at 1,000 ×g for 5 min. The supernatant was further centrifuged at 4,000 ×g for 10 min to remove cell debris. The resulting supernatant was centrifuged at 12,000 ×g for 15 min. The pellet was washed twice with prechilled (4℃) suspension buffer (20mM HEPES, 400mM sorbitol, 9mM KCl, 2mM EDTA, 6mM Na-AsA) and resuspended in 1mL of the same buffer to obtain the crude mitochondrial fraction.

### Mitochondrial purification (Percoll density gradient centrifugation)

A discontinuous Percoll gradient (Percoll reagent: Biotopped, Beijing, Cat. No. P1701H) was prepared by layering 2mL of 42%, 4mL of 35%, and 4mL of 23% percoll solutions (in 0.25 M sucrose) into a 13.2mL centrifuge tube (Beckman Coulter, Cat. No. 344059). The crude mitochondrial suspension (2mL) was carefully layered on top. The gradient was centrifuged at 120,000 ×g for 60 min at 4℃ using an ultracentrifuge (Beckman Optima XPN-100, USA). Mitochondria formed a faint yellow band between the 23% and 35% percoll layers, which was collected using a syringe. The mitochondrial fraction was washed three times with wash buffer (10mM Tricine, 251mM sucrose, 1mM EDTA, 5.67mM Na-AsA, 0.3% BSA) by centrifugation at 15,000 ×g for 15 min (final wash excluded BSA). The purified mitochondria were resuspended in suspension buffer.

### Mitochondrial quality assessment

#### Integrity and viability assays

Mitochondrial suspension was mixed with Janus Green B working solution at a 1:1 ratio. After staining at room temperature for 15–20 min, an aliquot of the stained mitochondria was placed on a glass slide, covered with a coverslip, and imaged using a Leica optical microscope (Model: M205 FA, SN: 6080940). Viable mitochondria appeared blue-green and exhibited an oval or elliptical morphology.

For fluorescence-based assessment, the mitochondrial suspension was mixed with MitoTracker Red staining solution at a 1:1 ratio, incubated for 15–20 min at room temperature, and similarly imaged under a fluorescence microscope (Olympus BX53). Viable mitochondria emitted red fluorescence. The enzymatic activity of cytochrome c oxidase (COX) in both crude mitochondria and purified mitochondria was measured using a commercial COX Activity Assay Kit (Shanghai hengyuan biological technology co., Ltd., Cat. No. HS064X-Pt), following the manufacturer’s instructions with minor adaptations. For specific details, refer to Yang et al. (2022) [25].

#### Purity analysis

Mitochondrial pellets were lysed with RIPA buffer (Beyotime, P0045) containing 1 mM PMSF (Solarbio, P0100) on ice for 20 min. Lysates were centrifuged at 14,000 ×g for 15 min, and protein concentrations were determined using a BCA assay kit (Solarbio, PC0020). Absorbance at 562 nm was measured with a Thermo Scientific microplate reader.

Protein samples (20 µg) were separated by 10% SDS-PAGE, transferred to PVDF membranes, and probed with antibodies against β-actin, RbcL, and AOX. HRP-conjugated secondary antibodies (1:10,000, Takara) and ECL reagents (Biosharp) were used for detection. Band intensities were quantified using ImageJ (NIH).

### Mitochondrial ultrastructure (TEM)

Purified mitochondria were fixed in 2.5% glutaraldehyde, post-fixed in 1% OsO₄, dehydrated in an ethanol-acetone series, and embedded in Epon812 resin. Ultrathin sections (60 nm) were stained with uranyl acetate and lead citrate and imaged using a Hitachi HT7700 transmission electron microscope.

### Mitochondrial membrane integrity

Membrane potential (ΔΨm) was assessed using the JC-1 kit (Beyotime, C2003S). Mitochondria were treated with CCCP (negative control) or left untreated, stained with JC-1, and analyzed via fluorescence microscopy (Olympus BX53). JC-1 monomers (indicative of depolarized mitochondria) were detected at excitation/emission (Ex/Em) wavelengths of 490/530 nm (green fluorescence), while JC-1 aggregates (indicative of intact membrane potential) emitted red fluorescence at Ex/Em 525/590nm. Images were analyzed using ImageJ, and Red/green fluorescence ratios were calculated.

### Inner membrane complex activity

To evaluate the functional activity of mitochondrial electron transport chain (ETC) complexes, two distinct inhibitors were employed: rotenone (0, 10, 20, 30, 40, 50µM) to inhibit Complex I, and malonate (0, 5, 10, 25, 50mM) to inhibit Complex II. Isolated mitochondria were incubated with each inhibitor at 25℃ for 30 min. Following incubation, the activities of Complex I and Complex II were measured using specific assay kits (SuzhouGraceBiotechnologyCo.,Ltd, Cat. No. G0845W for Complex I and G0846W for Complex II), according to the manufacturer’s protocols.

### Temperature-dependent mitochondrial activity

To investigate the time-dependent changes in mitochondrial activity, isolated mitochondria were stored at three laboratory temperatures: 25℃, 4℃, and − 20℃. Sampling intervals were designed as follows: 25℃ storage: Samples were collected at 1, 3, 5, 7, 9, and 11 h post-storage. 4℃ storage: Samples were collected at 1, 3, 5, 7, 9, and 11 days post-storage. − 20℃ storage: Samples were collected at 1, 10, 20, 30, 40, 50, and 60 days post-storage. Collected samples were stained with JC-1 dye and loaded into black 96-well plates. Fluorescence signals were measured using a Synergy H1M multifunctional microplate reader (BioTek Instruments, USA). The red-to-green fluorescence ratio was calculated for each sample to evaluate mitochondrial membrane potential stability. Experiments were performed in three independent replicates, and data are presented as mean ± standard deviation (SD).

### Data analysis

All statistical analyses were performed using SPSS 22.0 (Statistical Product and Service Solutions, SPSS Inc., Chicago, USA) and GraphPad Prism 8. Intergroup comparisons were conducted by one-way analysis of variance (ANOVA), followed by the least significant difference (LSD) post hoc test to assess specific differences. Statistical significance was defined as *P* < 0.05. Data are presented as mean ± standard deviation (SD), with error bars indicating SD in figures.

## Data Availability

No datasets were generated or analysed during the current study.
